# Limited prognostic role of routine serum markers (AP, CEA, LDH and NSE) in oligorecurrent prostate cancer patients undergoing PSMA-radioguided surgery

**DOI:** 10.1007/s00345-024-04948-9

**Published:** 2024-04-24

**Authors:** Gisa Mehring, Christina Steinbach, Randi Pose, Sophie Knipper, Daniel Koehler, Stefan Werner, Sabine Riethdorf, Gunhild von Amsberg, Francesca Ambrosini, Tobias Maurer

**Affiliations:** 1 Martini-Klinik Prostate Cancer Center, University Medical Center Hamburg-Eppendorf, Martinistraße 52, 20246 Hamburg, Germany; 2 Department of Urology, Vivantes Klinikum Am Urban, Berlin, Germany; 3 Department of Diagnostic and Interventional Radiology and Nuclear Medicine, University Medical Center Hamburg-Eppendorf, Hamburg, Germany; 4 Department of Tumor Biology, University Medical Center Hamburg-Eppendorf, Hamburg, Germany; 5 Department of Oncology, Hematology and Bone Marrow Transplantation With Section Pneumology, Hubertus Wald Tumorzentrum – University Cancer Center Hamburg (UCCH), University Medical Center Hamburg-Eppendorf, Hamburg, Germany; 6 IRCCS Ospedale Policlinico San Martino, Genoa, Italy; 7 Department of Urology, University Medical Center Hamburg-Eppendorf, Hamburg, Germany

**Keywords:** Biomarker, Biochemical recurrence, Salvage surgery, PET

## Abstract

**Introduction:**

We evaluated the prognostic role of pre-salvage prostate-specific membrane antigen–radioguided surgery (PSMA-RGS) serum levels of alkaline phosphatase (AP), carcinoembryonic antigen (CEA), lactate dehydrogenase (LDH), and neuron-specific enolase (NSE).

**Materials and methods:**

Patients who consecutively underwent PSMA-RGS for prostate cancer (PCa) oligorecurrence between January 2019 and January 2022 were selected. Biomarkers were assessed one day before surgery. Cox regression and logistic regression models tested the relationship between biochemical recurrence-free survival (BFS), 6- and 12-month biochemical recurrence (BCR), and several independent variables, including biomarkers.

**Results:**

153 consecutive patients were analyzed. In the univariable Cox regression analysis, none of the biomarkers achieved predictor status (AP: hazard ratio [HR] = 1.03, 95% CI 0.99, 1.01; *p* = 0.19; CEA: HR = 1.73, 95% CI 0.94, 1.21; *p* = 0.34; LDH: HR = 1.01, 95% CI 1.00, 1.01; *p* = 0.05; NSE: HR = 1.02, 95% CI 0.98, 1.06; *p* = 0.39). The only independent predictor of BFS was the number of positive lesions on PSMA PET (HR = 1.17, 95% CI 1.02, 1.30; *p* = 0.03).

The number of positive lesions was confirmed as independent predictor for BCR within 6 and 12 months (BCR < 6 months: odds ratio [OR] = 1.1, 95% CI 1.0, 1.3; *p* = 0.04; BCR < 12 months: OR = 1.1, 95% CI 1.0, 1.3; *p* = 0.04).

**Conclusion:**

The assessment of AP, CEA, LDH, and NSE before salvage PSMA-RGS showed no prognostic impact. Further studies are needed to identify possible predictors that will optimize patient selection for salvage PSMA-RGS.

**Supplementary Information:**

The online version contains supplementary material available at 10.1007/s00345-024-04948-9.

## Introduction

The widespread adoption of imaging techniques such as positron emission tomography (PET), using radiotracers targeting the prostate-specific membrane antigen (PSMA), has significantly improved the early detection of recurrent prostate cancer (PCa). This has proven to be particularly effective in identifying lesions in the pelvic region before systemic metastatic disease occurs, even at very low levels of prostate-specific antigen (PSA) [1–3].

Although the standard treatment for oligorecurrent hormone-sensitive prostate cancer (HSPC) usually consists of systemic combination treatment with androgen receptor-targeted agents (ARTA) and androgen deprivation therapy (ADT) [4–6], PSMA-radioguided surgery (RGS), which is still considered experimental, has been shown to be effective in removing PCa metastases and achieving consecutive PSA responses [7].

However, the greatest challenge lies in selecting the ideal candidate, as reliable biomarkers, nomograms or imaging techniques are not yet conclusive [8].

Recently, we attempted to evaluate the role of the European Association of Urology (EAU) risk groups for BCR and PSA kinetics (including PSA doubling time and PSA velocity) in the selection of patients for PSMA-RGS. According to their results, neither BCR risk groups nor PSA kinetics have an impact on the prediction of complete biochemical response, BCR-free and treatment-free survival in PSMA-RGS performed at low PSA values [8].

Thus, further guidance for patient selection by readily available biomarkers would be of great value. In the past, elevated serum levels of carcinoembryonic antigen (CEA), lactate dehydrogenase (LDH) and neuron-specific enolase (NSE) have already been described in the context of neuroendocrine dedifferentiation and aggressive-variant PCa [9]. According to the available literature, there is a possible correlation between elevated serum levels of NSE and prognosis in advanced PCa, particularly in the context of metastatic castration-resistant PCa (mCRPC) [10]. A recent meta-analysis found that elevated serum LDH levels are associated with an increased risk of mortality and progression in patients with “low volume” and “high volume” metastatic PCa [11]. The prognostic role of CEA is still controversial in PCa [12, 13]. Moreover, several clinical studies and a systematic review reported that alkaline phosphatase (AP) levels are associated with an increased risk of overall mortality and disease progression in patients with metastatic HSPC [14].

Thus, we aimed to assess the value of AP, CEA, LDH and NSE serum levels prior to PSMA-RGS in oligorecurrent PCa as potential tools for guiding treatment strategy and patient selection.

## Materials and methods

### Study cohort

Patients who consecutively underwent PSMA-RGS for PCa pelvic, retroperitoneal or soft tissue oligorecurrence on PSMA PET between January 2019 and January 2022 were selected from our prospectively collected institutional review board-approved database (institutional review board (2019-PS-09; PV7316), Hamburg, Germany). Patients were selected for PSMA-RGS according to previously published criteria [7].

All patients had undergone radical prostatectomy (RP) as primary treatment, and subsequently were diagnosed with biochemical recurrence (BCR) with one or more positive soft tissue or lymph node lesions on PSMA PET. AP, CEA, LDH and NSE serum values were assessed one day prior to salvage surgery.

Patients were excluded from the analysis in case of ADT within six months prior to PSMA PET or involvement in a prospective clinical trial (*n* = 25). PSMA-RGS was performed as previously described [7, 15, 16].

### Outcomes of interest

The rate of complete biochemical response (cBR, defined as PSA < 0.2 ng/ml) without any additional treatment was determined 2–16 weeks following PSMA-RGS. Furthermore, BCR-free survival (BFS) was assessed and calculated from the time of PSMA-RGS to the event (no cBR or PSA progression ≥ 0.2 ng/ml after cBR) or end of follow-up. Patients were censored on the date of last evidence of freedom from BCR or further treatment.

### Statistical analysis

Descriptive statistics included frequencies and proportions for categorical variables. Means, medians, and ranges were reported for continuously coded variables.

To test if there are significant differences in the ordinal distributions of the level of biomarkers across the different levels of the location of positive lesions, the non-parametric Kruskal–Wallis test was performed.

Univariable and multivariable Cox regression models tested the relationship between BFS and several variables, namely, age at PSMA-RGS (continuously coded), Gleason grade group at RP (I–II vs III–V), pN stage at RP (N0/NX vs N1), radiation therapy (RT) after RP prior to PSMA-RGS (no vs yes), time between initial RP and PSMA-RGS (continuously coded), PSA prior PSMA-RGS (continuously coded), number of PSMA PET positive lesions prior to PSMA-RGS (continuously coded), levels of biomarkers before PSMA-RGS (AP, CEA, LDH, NSE, all continuously coded). Predictors were selected among potential factors previously published and associated with oncological outcomes after PSMA-RGS [7]. These were included in the multivariable models if significantly associated with the outcome in the univariable analysis.

Moreover, considering the potential clinical impact on patient selection, univariable logistic regression models were performed to test whether biomarkers above the normal range could predict early BCR (no vs yes) within 6 or 12 months [17].

For all statistical analyses, R software environment for statistical computing and graphics (version 4.1.2) was used. All tests were two sided, with a level of significance set at *p* < 0.05.

## Results

Overall, 153 consecutive patients were analyzed.

Patients underwent PSMA-RGS at a median age of 66 years (IQR: 61, 70), and with a median PSA level of 0.6 ng/ml (IQR: 0.4, 1) (see Table 1; for initial patients’ characteristics see Supplementary Table 1).

**Table 1:** Patient’s characteristics at salvage PSMA-RGS

Parameter	No
Age at PSMA-RGS (yr), median (IQR)	66 (61, 70)
Time between RP and PSMA-RGS (months), median (IQR)	44 (23, 77)
PSA prior to PSMA-RGS (ng/ml), median (IQR)	0.6 (0.4, 1)
AP (U/l), median (IQR)*	65 (56, 81)
AP category (U/l), *n* (%)	
≤ 130	151 (99)
> 130 U/l	2 (1.3)
CEA (µg/l), median (IQR)**	0.8 (0.5, 1.5)
CEA category (µg/l), *n* (%)	
< 2.5 µg/l	143 (93)
≥ 2.5 µg/l	10 (7)
LDH (U/l)***	197 (176, 213)
LDH cathegory (U/l), *n* (%)	
< 250 U/l	142 (93)
≥ 250 U/l	11 (7)
NSE (µg/l)****	14.3 (12.1, 15.2)
NSE category (µg/l)	
< 18.3 µg/l	133 (86.9)
≥ 18.3 µg/l	16 (10.4)
Missing	4 (2.7)
No. of PSMA PET/CT positive lesions, median (IQR)	1 (1, 2)
PSMA PET/CT localization, *n* (%)	
Retrovesical/paravesical	27 (17.6)
Pelvic	79 (51.6)
Retroperitoneal	27 (17.6)
Equivocal	20 (13.2)
Lymph node yield at SLND, median (IQR)	15 (10, 23)
No. of pathologically positive lymph nodes, *n* (%)	2 (1, 4)
Location of positive lesions, *n* (%)	
Retrovesical/paravesical	25 (16.2)
Pelvic	67 (43.8)
Retroperitoneal	41 (26.8)
Negative	20 (13.2)
Follow-up (months)	13 (6, 24)

*IQR* interquartile range, *PET* positron emission tomography, *PSA* prostate-specific antigen, *PSMA* prostate-specific membrane antigen, *PSMA-RGS* PSMA-radioguided surgery, *RP* radical prostatectomy, *AP* alkaline phosphatase, *CEA* carcinoembryonic antigen, *LDH* lactate dehydrogenase, *NSE* neuron-specific enolase

*AP regular range: 4–130 U/l

**CEA regular tange: < 2.5 (smoker < 5) µg/l

***LDH regular range: < 250 U/l

****NSE regular range: < 18.3 µg/l

The median values of AP, CEA, LDH, and NSE were 65 U/l (IQR: 56, 81), 0.8 µg/l (IQR: 0.5, 1.5), 197 U/l (IQR: 176, 213), and 14.3 µg/l (IQR: 12.1, 15.2), respectively (Table 1). Only in two (1.3%), ten (7%), eleven (7%) and 16 (10.4%) patients AP, CEA, LDH and NSE values were above the regular ranges. No significant correlation between the distribution of the biomarkers and the location of positive lesions was found (Supplementary Table 4).

### Oncological outcomes

At 2–16 weeks after PSMA-RGS, 84 (55%) patients reached complete biochemical response. At a median follow-up of 13 months (IQR: 6, 24), 88 (57.5%) patients experienced BCR. The rate of BFS at 12 months was 43.3%. Within 6 and 12 months, 73 (48%) and 78 (51%) patients experienced BCR, respectively. The oncological outcomes of each patient according to the level of biomarkers assessed before PSMA-RGS are reported in Supplementary Fig. 1.

In the univariable Cox regression analysis, none of the biomarkers achieved predictor status, even coding the biomarkers as categorical variable with cut-off (AP: hazard ratio [HR] = 1.03, 95% confidence interval [CI]: 0.99, 1.01; *p* = 0.19; CEA: HR = 1.73, 95% CI 0.94, 1.21; *p* = 0.34; LDH: HR = 1.01, 95% CI 1.00, 1.01; *p* = 0.05; NSE: HR = 1.02, 95% CI 0.98, 1.06; *p* = 0.39) (Table 2). The only independent predictor of BFS was the number of positive lesions (HR = 1.17, 95% CI 1.02, 1.30; *p* = 0.03) (Table 2). PSA level performed prior to PSMA-RGS did not achieve predictor status (HR = 1.09, 95% CI 0.98, 1.21; *p* = 0.12) (Table 2). The number of positive lesions (HR = 1.19, 95% CI 1.04, 1.38; *p* = 0.01) was also confirmed as predictor of BFS in the multivariable model (Supplementary Table 5).

**Table 2:** Univariable Cox regression models predicting biochemical recurrence-free survival

Parameter	Univariable Cox regression model			
	HR	CI 2.5%	CI 97.5%	*p*-value
Age at surgery	0.98	0.95	1.01	0.12
Gleason Grade group at RP				
I–II	Ref			
III–V	1.62	0.96	2.76	0.07
pN stage at RP				
pN0pNx	Ref			
pN1	1.19	0.69	2.06	0.53
RT after RP				
No	Ref			
Yes	1.07	0.70	1.63	0.76
Time from RP to PSMA-RGS, months	0.99	0.99	1.01	0.19
No. of PSMA PET-positive lesions	1.17	1.02	1.30	0.03***
PSA prior PSMA-RGS (ng/ml)*	1.09	0.98	1.20	0.12
AP (U/l)*	1.03	0.99	1.01	0.19
CEA (µg/l)*	1.07	0.94	1.21	0.34
LDH *(*U/l)*	1.01	1.00	1.01	0.05
NSE (µg/l)*	1.02	0.98	1.06	0.39
CEA (µg/l)**				
< 2.5 µg/l	Ref			
≥ 2.5 µg/l	2.37	0.63	11.3	0.20
LDH (U/l)**				
< 250 U/l	Ref			
≥ 250 U/l	2.74	0.76	12.9	0.15
NSE (µg/l)**				
< 18.3 µg/l	Ref			
≥ 18.3 µg/l	1.69	0.59	5.22	0.30

* prior to PSMA-RGS, continuously coded, **evaluated as categorical variable (within or above normal limits), AP as categorical variable was not considered due to the low number of events above normal limits, ***tested significant in univariate analysis (*p* < 0.05)

*CI* confidence interval, *HR* hazard ratio, *PET* positron emission tomography, *PSA* prostate-specific antigen, *PSMA* prostate-specific membrane antigen, *PSMA-RGS* PSMA-radioguided surgery, *Ref.* reference, *RP* radical prostatectomy, *RT* radiotherapy, *AP* Alkaline phosphatase, *CEA* carcinoembryonic antigen, *LDH* Lactate dehydrogenase, *NSE* neuron-specific enolase

According to the univariable logistic regression analysis, none of the biomarkers above the normal range achieved the role of predicting BCR within 6 and 12 months (Supplementary Tables 2 and 3).

The number of positive lesions was confirmed as the only independent predictor for BCR within 6 and 12 months (BCR < 6 months: odds ratio [OR] = 1.1, 95% CI 1.0, 1.3; *p* = 0.04; BCR < 12 months: OR = 1.1, 95% CI 1.0, 1.3; *p* = 0.04) (Supplementary Table 2 and 3).

## Discussion

In recent years, the widespread use of PSMA PET in both, staging and restaging, has sparked a strong interest in targeted imaging and individualized treatment strategies [1, 18]. This is particularly evident for open or robot-assisted PSMA-RGS as a patient-specific salvage treatment for PCa recurrence [19]. This technique has proven to be oncologically safe, as it effectively delays the start of systemic treatment in selected patients [7].

However, PSMA-RGS should still be considered experimental despite its encouraging results. One of the key requirements is the definition of clear criteria for patient selection. There is an unmet need to define the ideal candidates who would benefit most from salvage PSMA-RGS [7]. According to our previous analysis, patients with low PSA values at salvage surgery and a low number of PSMA-PET–avid lesions, ideally located in the pelvis, could derive the greatest benefit from PSMA RGS [7, 20].

In our analysis none of the tested serum biomarkers (AP, CEA, LDH and NSE) reached predictor status for BCR after PSMA-RGS on univariate analysis. The same results were reported in the additional analysis testing the role of biomarkers above the regular range in predicting BCR within 6 and 12 months. We hypothesize that the most important factor affecting the robustness of the models is the limited number of patients who had biomarkers beyond the regular range, and within this subgroup, those who experienced BCR.

In general, the literature exploring the role of biomarkers in oncologic outcomes of PCa is controversial. AP is one of the most widely studied markers which was found to be associated with OS and the risk of disease progression, but not with cancer-specific mortality in both, low and high volume metastatic HSPC [14, 19]. Nevertheless, Tsuzuki et al. developed a prognostic model including Gleason pattern 5, AP and LDH as predictors for a shorter time to CRPC and thus a poorer prognosis among patients with low-volume HSPC [21].

However, our results may not be directly comparable to the existing literature since we focused on patients with low metastatic burden, and we could hypothesize that biomarker values may increase in correlation with the extent of metastasis.

Most studies supporting the use of AP as a prognostic tool in the context of PCa treatment refer to metastatic CRPC [22, 23].

Similar considerations could be made for LDH, which is generally used as a marker of cancer burden in oncology. According to a recent systematic review and meta-analysis, LDH was associated with OS and PFS in patients with metastatic PCa [24]. However, most of the included studies were retrospective and had limited sample sizes, exclusion of patients with conditions that could affect LDH was not always specified, and LDH was categorized in the analysis with cutoff values that were not consistent within the original studies. Thus, caution should be taken when interpreting these results. Similar data were reported in the systematic review and meta-analysis by Mori et al. [11]. Nevertheless, only 14.3% of patients had HSPC, while the larger proportion was CRPC (85.7%).

Lastly, comparable thoughts should be given to NSE which could increase in CRPC compared to HSPC in the case of neuroendocrine transdifferentiation under androgen deprivation therapy [25]. Most of the studies reporting an association between increased NSE levels and poor prognosis are related to CRPC [10, 26]. Despite consistently incorporating CRPC, some studies did not confirm the prognostic role of NSE [25, 26].

Regarding CEA, the existing literature is limited to historical and small cohorts [27–29]. CEA was found to predict overall survival but not progression in patients with “anaplastic” small-cell prostate carcinoma during platinum-based chemotherapy [30]. Consistent with our findings, Nan et al. found that CEA levels provided prognostic information for breast cancer, gastric cancer and pancreatic cancer, but not for PCa [12].

However, taking all these aspects together and considering that this is the only study available in the literature on this topic, we believe that our results provide valuable insights into the tools that should or should not be considered in patient selection for PSMA-RGS.

In the current landscape, the most suitable candidates for salvage PSMA-RGS treatment are still patients with oligorecurrent disease, low PSA values and a limited number of positive lesions on PSMA-PET/CT, as shown in our previously published cohort.

Some limitations of our analysis should be mentioned. First, the study is constrained by a limited follow-up period. Due to limited follow-up in our cohort, it is not possible to have further insight on a stronger intermediate endpoint such as clinical progression. Moreover, the sample size is limited, and the number of events is low, thus the results of regression analysis should be interpreted with caution. Specifically, multivariable Cox regression models would have been prone to overfitting.

In conclusion, refining patient selection criteria is the crucial point to establishing the role of PSMA-RGS as a viable option for the treatment of recurrent PCa. In this context, while our results do not offer immediate or major clinical application, they do provide further insight into ongoing efforts to refine the ideal candidate. In the current scenario, we believe that all data, including negative data, has a valuable role to play in improving knowledge.

In the future evolving landscape, circulating tumour cells (CTCs) should be mentioned among the biomarkers that could play a prognostic role. In a pilot study, it was found that clinical and pathological outcomes are worse when CTCs can be detected in the blood preoperatively [31]. These results have laid the foundation for a prospective study (“BioPoP: Identification of Predictive Biomarkers” (NCT04324983)) investigating the predictive role of biomarkers in the selection of the ideal candidate for salvage PSMA-RGS.

## Conclusion

The preoperative assessment of biomarkers, namely AP, CEA, LDH and NSE, before salvage PSMA-RGS for oligorecurrent PCa showed no prognostic significance. Further studies are needed to identify effective prognostic tools to optimize patient selection for salvage PSMA-RGS.

## Supplementary Information

Below is the link to the electronic supplementary material.

Supplementary file1 (DOCX 18 KB)

Supplementary file2 (DOCX 17 KB)

Supplementary file3 (DOCX 17 KB)

Supplementary file4 Supplementary Figure 1: Scatter plot showing the oncological outcomes according to the levels of preoperative biomarkers in 153 consecutive patients who underwent salvage PSMA-radioguided surgery. The scatter plots depict the levels of the four biomarkers over time, with each point color-coded based on the presence or absence of BCR (JPG 100 KB)

Supplementary file5 (DOCX 16 KB)

Supplementary file6 (DOCX 17 KB)

## Data Availability

Upon reasonable request raw data can be made available for research purposes in accordance with applicable data protection regulations.

## Abbreviations

ADT: Androgen deprivation therapy
AP: Alkaline phosphatase
ARTA: Androgen receptor-targeted agents
BCR: Biochemical recurrence
BFS: Biochemical recurrence free survival
cBR: Complete biochemical response
CEA: Carcinoembryonic antigen
CT: Computed tomography
HSPC: Hormone-sensitive prostate cancer
LDH: Lactate dehydrogenase
NSE: Neuron specific enolase
PCa: Prostate cancer
PET: Positron emission tomography
PSMA: Prostate-specific membrane antigen
RGS: Radioguided surgery
^99m^Tc: ^99M^Technetium
IQR: Interquartile range
