# Supplementary material for: Limited prognostic role of routine serum markers (AP, CEA, LDH and NSE) in oligorecurrent prostate cancer patients undergoing PSMA-radioguided surgery
Source: World J Urol. 2024 Apr 24;42(1):256. doi: 10.1007/s00345-024-04948-9 (PMC11043188; doi:10.1007/s00345-024-04948-9)
Supplement: Supplementary file 1 — Supplementary file1 (DOCX 18 KB) [file 345_2024_4948_MOESM1_ESM.docx]

Supplementary Table 1 – Initial patients´ characteristics.

| **Parameter** | **No.** |
| --- | --- |
| **Number of patients, n (%)** | 153 |
| **Year of initial RP, median (IQR)** | 2017 (2014, 2019) |
| **PSA at RP (ng/ml), median (IQR)** | 9 (6, 15) |
| **pT stage at RP, n (%)** |  |
| pT2 | 45 (29.4) |
| pT3a | 48 (31.4) |
| pT3b | 45 (29.4) |
| pT4 | 1 (0.6) |
| Missing | 14 (9.2) |
| **Gleason grade group, n (%)** |  |
| I | 5 (3.3) |
| II | 35 (22.8) |
| III | 62 (40.5) |
| IV | 14 (9.2) |
| V | 23 (15) |
| Missing | 14 (9.2) |
| **pN stage at RP, n (%)** |  |
| pN0 | 103 (67.3) |
| pN1 | 28 (18.3) |
| pNX | 22 (14.4) |
| **Lymph node yield at RP, median (IQR)** | 13 (7, 18) |
| **No. of positive lymph nodes at RP, n (%)** |  |
| 0 | 100 (65.4) |
| 1 | 14 (9.1) |
| 2 | 6 (3.9) |
| 3 | 3 (2) |
| 4 | 2 (1.3) |
| Missing | 28 (18.3) |
| **Surgical margin status, n (%)** |  |
| R0 | 99 (65) |
| R1 | 37 (24) |
| Missing | 17 (11) |
| **RT after RP, n (%)** |  |
| No RT | 83 (54) |
| RT | 70 (46) |

cBR = complete biochemical response (defined as PSA < 0.2 after 2-16 weeks after RGS); IQR= interquartile range; PSA = prostate-specific antigen; RP = radical prostatectomy; RT = radiation therapy.
