# Supplementary material for: Limited prognostic role of routine serum markers (AP, CEA, LDH and NSE) in oligorecurrent prostate cancer patients undergoing PSMA-radioguided surgery
Source: World J Urol. 2024 Apr 24;42(1):256. doi: 10.1007/s00345-024-04948-9 (PMC11043188; doi:10.1007/s00345-024-04948-9)
Supplement: Supplementary file 2 — Supplementary file2 (DOCX 17 KB) [file 345_2024_4948_MOESM2_ESM.docx]

Supplementary Table 2: Univariable regression analysis to test predictors for BCR < 6 months.

| **Parameter*** | OR | 95% CI | p-value |
| --- | --- | --- | --- |
| **CEA** |  |  |  |
| <2.5 µg/l | Ref. | — |  |
| ≥2.5 µg/l | 2.7 | 0.7, 13.0 | 0.2 |
| **LDH** |  |  |  |
| <250 U/l | Ref. | — |  |
| ≥250 U/l | 3.16 | 0.87, 14.9 | 0.10 |
| **NSE** |  |  |  |
| <18.3 µg/l | Ref. | — |  |
| ≥18.3 µg/l | 1.9 | 0.7, 6.1 | 0.2 |
| **PSA at RGS (ng/ml)** | 1.1 | 0.9, 1.4 | 0.4 |
| **Number of positive lesions** | 1.1 | 1.0, 1.3 | 0.04 |

OR = odd ratio; CI = confidence interval; Ref = reference; AP = alkaline phosphatase; CEA = carcinoembryonic antigen; LDH = lactate dehydrogenase; NSE = neuron-specific enolase; PSA = prostate-specific antigen; RGS = radioguided-surgery.

*AP not tested due to the low number of patients with biomarker above the regular range.
