# Supplementary figures and images for: Limited prognostic role of routine serum markers (AP, CEA, LDH and NSE) in oligorecurrent prostate cancer patients undergoing PSMA-radioguided surgery
Source: World J Urol. 2024 Apr 24;42(1):256. doi: 10.1007/s00345-024-04948-9 (PMC11043188; doi:10.1007/s00345-024-04948-9)

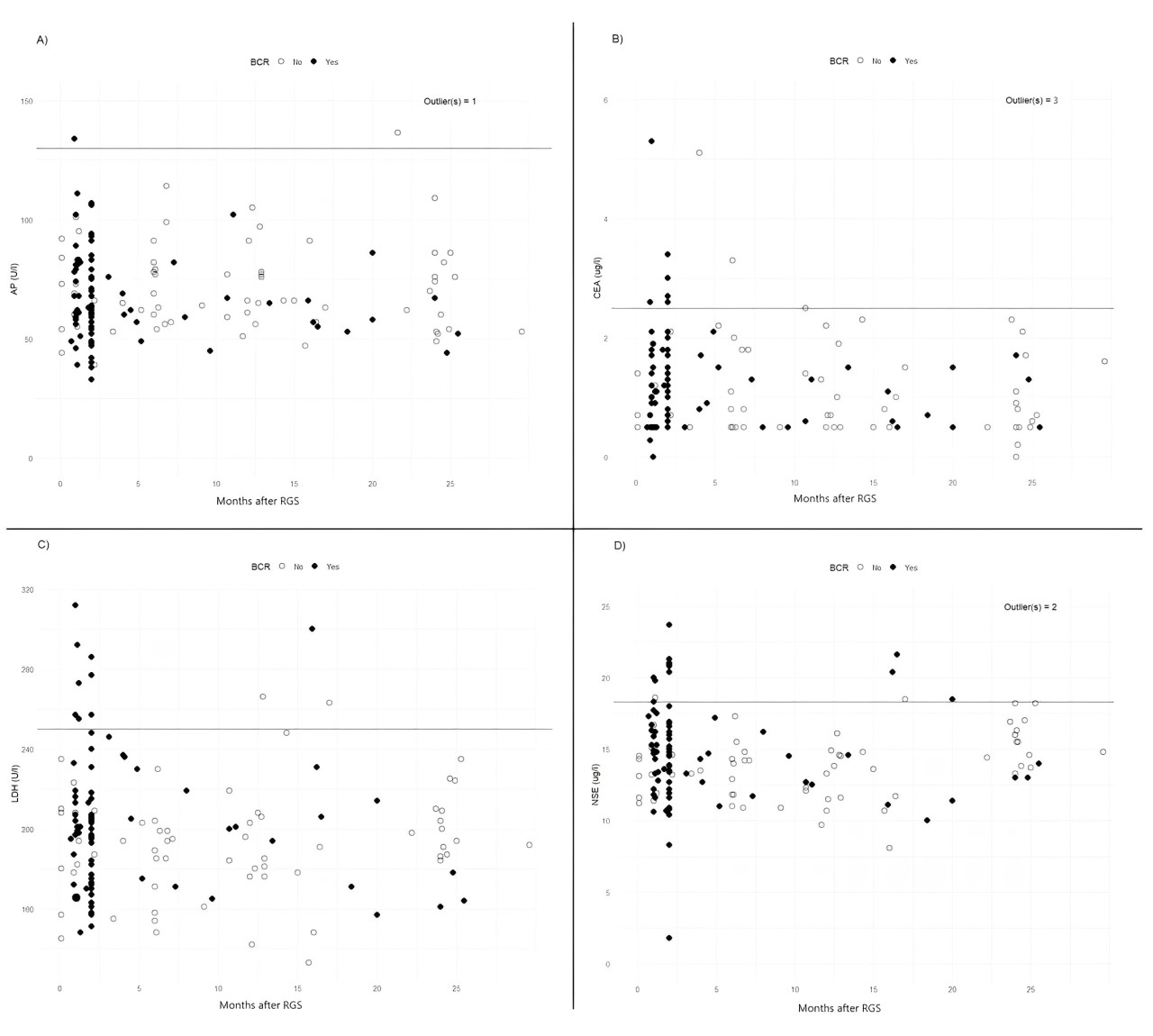

Supplement: Supplementary file 4 — Supplementary file4 Supplementary Figure 1: Scatter plot showing the oncological outcomes according to the levels of preoperative biomarkers in 153 consecutive patients who underwent salvage PSMA-radioguided surgery. The scatter plots depict the levels of the four biomarkers over time, with each point color-coded based on the presence or absence of BCR (JPG 100 KB) [file 345_2024_4948_MOESM4_ESM.jpg]
