# Supplementary material for: Limited prognostic role of routine serum markers (AP, CEA, LDH and NSE) in oligorecurrent prostate cancer patients undergoing PSMA-radioguided surgery
Source: World J Urol. 2024 Apr 24;42(1):256. doi: 10.1007/s00345-024-04948-9 (PMC11043188; doi:10.1007/s00345-024-04948-9)
Supplement: Supplementary file 5 — Supplementary file5 (DOCX 16 KB) [file 345_2024_4948_MOESM5_ESM.docx]

Supplementary Table 4: Correlation between the distribution of the biomarkers and the location of positive lesions

|  | **Location of positive lesions** | | | |  |
| --- | --- | --- | --- | --- | --- |
|  | Retrovesical/  paravesical | Pelvic | Retroperitoneal | Negative | P value |
| AP (U/l), median (IQR) * | 61 (56, 74) | 66 (55.5, 77.5) | 67.5 (57, 94.2) | 68 (57, 81) | 0.3 |
| CEA (µg/l), median (IQR) * | 1.1 (0.5, 1.8) | 0.6 (0.5, 1.3) | 1 (0.5, 1.5) | 0.9 (0.5, 1.6) | 0.3 |
| LDH (U/l), median (IQR) * | 203 (186, 213) | 195 (171, 211.5) | 195 (173.8, 230.2) | 195 (182, 206) | 0.5 |
| NSE (µg/l), median (IQR) * | 14.3 (11.8, 15.6) | 14 (12.3, 16.2) | 14.6 (12.3, 16.3) | 14.4 (12, 15.6) | 0.9 |

* prior to PSMA-RGS, continuously coded

AP= Alkaline phosphatase, CEA= Carcinoembryonic antigen, LDH= Lactate dehydrogenase, NSE= Neuron-specific enolase.
