# Supplementary material for: Limited prognostic role of routine serum markers (AP, CEA, LDH and NSE) in oligorecurrent prostate cancer patients undergoing PSMA-radioguided surgery
Source: World J Urol. 2024 Apr 24;42(1):256. doi: 10.1007/s00345-024-04948-9 (PMC11043188; doi:10.1007/s00345-024-04948-9)
Supplement: Supplementary file 6 — Supplementary file6 (DOCX 17 KB) [file 345_2024_4948_MOESM6_ESM.docx]

Supplementary Table 5: Multivariable Cox regression models predicting biochemical recurrence-free survival.

|  | Multivariable Cox regression model | | | |
| --- | --- | --- | --- | --- |
| **Parameter** | **HR** | **CI 2.5%** | **CI 97.5%** | **p-value** |
| **No. of PSMA PET-positive lesions** | **1.19** | **1.04** | **1.38** | **0.01** |
| PSA prior PSMA-RGS (ng/ml) * | 0.99 | 0.85 | 1.16 | 0.91 |
| AP (U/l) * | 1.00 | 0.99 | 1.01 | 0.28 |
| CEA (µg/l) * | 1.29 | 0.89 | 1.87 | 0.18 |
| LDH (U/l) * | 1.00 | 0.99 | 1.01 | 0.53 |
| NSE (µg/l) * | 1.01 | 0.96 | 1.07 | 0.59 |

* prior to PSMA-RGS, continuously coded

CI = confidence interval; HR = hazard ratio; PET = positron emission tomography; PSA = prostate-specific antigen; PSMA = prostate-specific membrane antigen; PSMA-RGS = PSMA-radioguided surgery; Ref. = reference; RP = radical prostatectomy; RT = radiotherapy; AP= Alkaline phosphatase, CEA= Carcinoembryonic antigen, LDH= Lactate dehydrogenase, NSE= Neuron-specific enolase.
